# Citrus Aurantium and caffeine complex versus placebo on biomarkers of metabolism: a double blind crossover design

**DOI:** 10.1186/s12970-019-0271-1

**Published:** 2019-02-06

**Authors:** Brian Kliszczewicz, Emily Bechke, Cassie Williamson, Zackery Green, Paul Bailey, John McLester, Cherilyn McLester

**Affiliations:** Department of Exercise Science and Sport Management, Kennesaw State University, Kennesaw, GA USA

**Keywords:** P-synephrine, Metabolism, Caffeine, Glucose, Insulin

## Abstract

**Backgrouond:**

The purpose of this study was to examine resting the metabolic response to the ingestion of a complex containing Citrus Aurantium + Caffeine (CA + C) and if its consumption influences metabolic recovery following a high-intensity anaerobic exercise bout in habitual caffeine users.

**Methods:**

Ten physically active males (25.1 ± 3.9 years; weight 78.71 ± 9.53 kg; height 177.2 ± 4.6 cm; body fat 15.5 ± 3.13%) participated in this study. This study was performed in a double-blind, randomized crossover fashion consisting of two exhaustive exercise protocols. On each visit the participants consumed either a CA + C (100 mg of CA and 100 mg of C) or placebo (dextrose) capsule. After consumption, participants were monitored throughout a 45-min ingestion period, then completed a repeated Wingate protocol, and were then monitored throughout a 45-min recovery period. Metabolic function was measured through blood glucose, plasma insulin, plasma triglycerides, and plasma catecholamines: epinephrine (E) and norepinephrine (NE). Biomarkers were taken at four different time points; Ingestion period: baseline (I1), post-ingestion period (I2); Recovery period: immediately post-exercise (R1), post-recovery period (R2).

**Results:**

A repeated measures ANOVA revealed significant time-dependent increases in plasma E and NE at I2 only in the CA + C trial (*p* < 0.05), and a significant decrease in blood glucose at I2 in the PLA trial (p < 0.05); however, no meaningful changes in glucose was observed following CA + C ingestion. No changes in insulin or triglycerides were observed during the ingestion period. No trial-dependent differences were observed in the Recovery period. All biomarkers of metabolic recovery were equivalent when evaluating R1 v R2. Participants recovered in a similar time-dependent manner in all markers of metabolism following the PLA and CA + C trials.

**Conclusion:**

The findings of this study suggested that normal recommended dosages of 100 mg CA + 100 mg C is sufficient to promote glucose sparing at rest, with modest increases in SNS activity; however, the individual role of CA or C in this response cannot be determined.

## Introduction

Thermogenic supplements have long been of interest to those seeking to improve body composition and performance enhancement. Recently within the field of health and fitness, the supplement complex of Citrus Aurantium and Caffeine (CA + C) has been purported to assist in weight loss through a number of physiological responses, such as increased energy expenditure, increased oxidation of lipids, and enhanced sympathetic drive [1, 2]. It is important to note that information regarding this complex is relatively limited when evaluating metabolic responses and should be further studied to determine the efficacy of the claim that it can assist in improving metabolic function.

Individually, the primary components of the CA + C complex have demonstrated favorable responses in caloric energy expenditure. For instance, the active component of the Citrus Aurantium, p-synephrine, enhances sensitivity to beta-3 receptors and improves lipolysis and resting metabolism [1, 3, 4]. Whereas caffeine has been widely demonstrated to increase various markers of exercise performance in conjunction to energy expenditure [5, 6]. Few studies have fully examined the combined CA + C complex in an isolated form; however, a recent study by Ratamess et al. [2] showed improved lipolysis and energy expenditure during rest with increases in fat oxidation following resistance-based exercise. These participants were not identified as habitual users of caffeine, hence it is unknown if the CA + C complex would be as effective in individuals that are habitual consumers.

Caffeine is perhaps one of the most widely utilized over the counter supplements in the fitness industry [6, 7]. Therefore, it is likely that the target population of the CA + C complex (i.e. athletes, fitness enthusiasts, etc.) would be habitual caffeine consumers. With this in mind, it has been demonstrated that habitual caffeine consumption leads to decreases in the sensitivity to a regular dosage of caffeine [8–11], (e.g. 100 mg). Each individual component, Citrus Aurantium and caffeine, primarily work through beta-receptor function and consequently influence downstream metabolic activity such as the regulation of blood glucose, insulin, triglycerides, and catecholamines. A decrease in caffeine responsiveness in habitual consumers may result in a less robust metabolic response than those reported in earlier studies [2]. Therefore, the purpose of this study was to examine the resting metabolic profile before and after CA (100 mg) + C (100 mg) consumption. Additionally, we aimed to evaluate post exercise metabolic recovery following an exhaustive exercise protocol.

## Methods

### Participants

All testing procedures and protocols were approved by the Universities’ Institutional Review Board prior to any data collection. Fourteen apparently healthy males that habitually consumed caffeine were recruited for this study. Habitual caffeine consumption was defined as ≥ one 95 mg serving per day, at least 4 days a week. Individuals who frequently consumed ≥300 mg/day of caffeine were excluded from the study. Physical activity inclusion criteria required all individuals to take part in no less than three-days of aerobic training and two-days of resistance training every week for a minimum of six months. Participants were recruited by word of mouth from the nearby metropolitan region. Prior to participation, all subjects were provided with procedures and made aware of risks associated with the study and the informed consent was signed. A health history questionnaire (HHQ) was administered in order to ensure that participants were able to take part in vigorous physical activity without medical clearance as characterized by the guidelines provided by American College of Sports Medicine [12]. Individuals reporting of any orthopedic conditions, cardiovascular, pulmonary, or metabolic disease were removed from the study. Participants were requested to wear comfortable clothing, to arrive fasted for a minimum of four-hours, avoid exercise and alcohol for 24-h, and refrain from caffeine usage for 12-h preceding each session to ensure its clearance from the blood [11].

### Experimental design

This study was performed in a double-blind, placebo-controlled, randomized crossover design where only one investigator knew the identification of the supplementation; this investigator did not engage in the gathering or analysis of data. Participants were requested to attend the Exercise Physiology Lab on two separate occasions, with the second visit taking place within three to nine days after the first visit. All visits were performed between 5 and 7 am. The initial visit comprised of obtaining the informed consent, HHQ, and anthropometric measures. Participant height (cm) and weight (kg) were gathered using an electronic physicians scale (Tanita WB 3000, Arlington Heights, IL). Body fat (BF%) was gathered via dual-energy x-ray absorptiometry scan (GE Lunar iDXA, Madison, WI) during the first visit using manufacturer’s guidelines and recommendations.

**Fig. 1:**
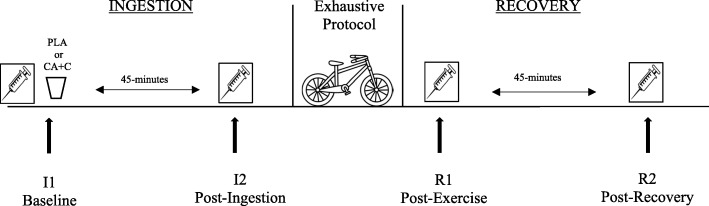
Study Design

The remaining two visits can be described in two overarching sections; the ingestion period with pre and post time points (I1 & I2) and the recovery period with pre and post time points (R1 & R2). The ingestion period consisted of baseline measures (I1), which included venipuncture, followed by the consumption of either the supplement (CA + C) or placebo (PLA). The 45-min ingestion period was initiated after the participants consumed CA + C or PLA. Upon the completion of the ingestion period, a post-ingestion venipuncture was performed (I2). Participants then performed a standardized warm-up prior to initiating the anaerobic exhaustive exercise protocol. Immediately following the exercise protocol a post-exercise venipuncture was performed (R1) and then the 45-min recovery period was initiated. At the end of this recovery period the final venipuncture was taken (R2). The study design can be seen in Fig. 1.

### Exhaustive exercise protocol

Upon the completion of the 45-min ingestion period, participants were allotted a seven-and-a-half minute warm up on a Monark ergometer (Monark 828E Ergomedic Test Cycle, Vansbro, Sweden) while pedaling between 50 and 100 rpm at a resistance of 1.5 kp. Participants were immediately walked to an electronically braked cycle ergometer (Sport Excalibur, Lode BV, Groningen, The Netherlands), where the bike was adjusted to the appropriate settings in order to ensure the knee was at a slight bend at the bottom of the revolution. Bike settings were repeated for both trials. Following the appropriate adjustments, participants feet were strapped into the pedals and the protocol was initiated. The start of the exhaustive exercise protocol comprised of a one-minute warm-up period performed at 50 W with a rolling start into the Wingate test. Each Wingate test was 30-s in duration and participants were encouraged to pedal at their maximal effort against a resistance of 0.80 Nm/kg [13]. There was a total of three Wingate tests performed with a two-minute active recovery period between each test. The active recovery was a self-selected pedal rate against a resistance of 50 W and a rolling start into the subsequent Wingate test. At the completion of the last Wingate test, participants were walked to a separate room to undergo a post exercise venipuncture and to begin the recovery measures (R1-R2). Pre-testing protocols on the electronically braked cycle ergometer followed manufacturer guidelines.

### Blood collection and analysis

Blood draws were collected via the antecubital vein by a trained phlebotomist during four-time points throughout the study: I1, I2, R1, R2 (Fig. 1). Each draw obtained six milliliters of blood using lithium heparin tubes and were inverted based on the manufactures’ recommendations prior to centrifugation. Samples were centrifuged at 2500 rpm for 15-min, then aliquoted and stored in a − 80 °C freezer until subsequent assay analysis. Plasma samples were assayed using commercially available ELISA kits for Insulin (Alpco, Salem, New Hampshire), Triglycerides (Infinity™, Thermo Scientific, Lexington, Massachusetts), E and NE (Abnova, Taoyuan City, Taiwan). In order to account for the plasma volume shifts following the exercise bout, all E and NE samples were normalized by using the established protocols of Dill and Costill [14]. Hematocrit (Hct) and hemoglobin (Hb) were collected via finger sticks at each venipuncture time point (Alere Hemopoint 2). Blood glucose (GLU) was measured using a Medtronic Contour glucometer (Bayer, Pittsburgh, PA) via finger stick. The procedures and findings of plasma catecholamines were previously reported and permissions granted by the publishing Journal [15].

### Supplement preparation

Citrus Aurantium and caffeine powder were purchased from Blackburn distributions (Caffeine powder, Blackburn distributions limited, Nelson Lancashire, England; Citrus Aurantium powder, Blackburn distributions limited, Nelson Lancashire, England). The PLA contained 200 mg of dextrose, whereas the supplement contained a combination of CA (100 mg) and C (100 mg). Each component was measured using an electronic supplement scale and encapsulated in green, non-translucent, size zero gelatin capsules.

### Statistical analysis

All data were analyzed using the statistical software package SPSS (SPSS, Version 24 for Mac, Chicago, IL). In order to assess changes in time within trial (CA + C/PLA) repeated measures analysis of variance (ANOVA) were run (I1 vs I2 and R1 vs R2) in the metabolic biomarkers glucose, insulin, and triglycerides. Significance for all statistical analyses was set at ≤0.05. The data is presented as the mean ± standard deviation (SD). In order to determine the effect size, the recommend guidelines of Quintana were used. [16]. Thresholds for effect size were the following; a small (< 0.25) moderate (0.50), and large effect (0.90).

## Results

Of the fourteen participants who volunteered for the study, four were removed due to adverse reactions to the phlebotomy procedure (i.e. vaso-vagal reactions) (*n* = 2), pain brought on by brochiospasm (*n* = 1), and trial monitoring issues (n = 1). Therefore, a total of ten physically active males completed the study. Participant characteristics can be seen in Table 1.

**Table 1:** Participant Characteristics (*N* = 10)

Characteristic	Mean **±** SD
Age (y)	25.1 ± 3.8
Height (cm)	177.2 ± 4.6
Weight (kg)	78.8 ± 9.4
Body Fat (%)	15.5 ± 3.0
Caffeine/day (mg)	209 ± 95.5

### Insulin

**Fig. 2:**
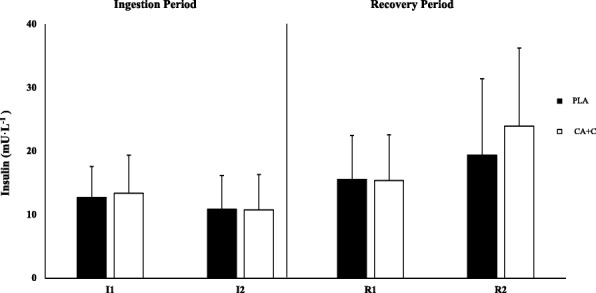
Plasma Insulin. Plasma insulin is presented as Means ± SD and expressed in International Units (mU·L^− 1^). * = significantly different from I1. + = Significantly different from PLA. # = Significantly different from R1

Repeated measures ANOVA showed no main effect between the trials in insulin (*p* = 0.198) while no significant time differences were observed when comparing I1 to I2 during the ingestion periods for PLA (*p* = 0.151) or for CA + C (*p* = 0.061) or -R1 to R2 during the recovery periods (*p* = > 0.999, *p* = 0.339), respectively (Fig. 2).

### Glucose

**Fig. 3:**
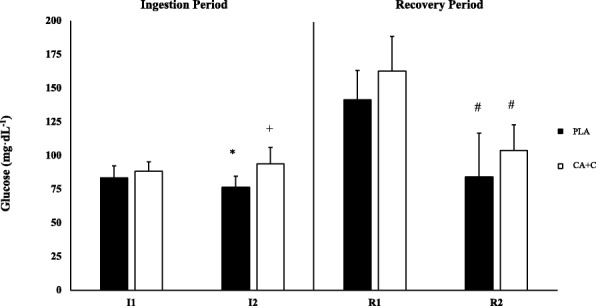
Blood Glucose. Blood glucose is presented as Means ± SD and expressed in mg/dL. * = significantly different from I1. + = Significantly different from PLA. # = Significantly different from R1

The repeated measures ANOVA showed a main trial effect (*p* = 0.045) in blood glucose. Specifically, I2 PLA was significantly lower when compared to I2 CA + C (*p* < 0.001, Cohen’s d 1.701). When evaluating PLA I1 vs I2 a significant decrease was observed (*p* = 0.04, Cohen’s d 0.839), while a no change in concentration was seen in the CA + C trial (*p* = 0.714). Both trials observed significant reductions in blood glucose when comparing R1 to R2 (*p* = 0.01, Cohen’s d 2.078; *p* < 0.001, Cohen’s d 2.586), respectively (Fig. 3).

### Triglycerides

**Fig. 4:**
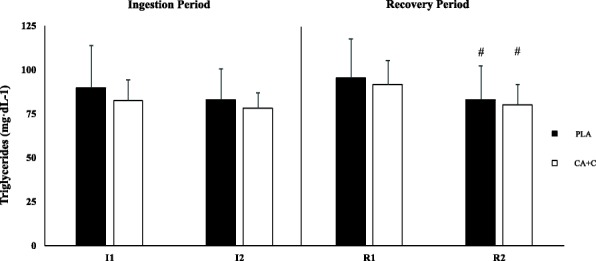
Plasma Triglycerides. Plasma triglycerides are presented as Means ± SD and expressed in mg/dL. * = significantly different from I1. + = Significantly different from PLA. # = Significantly different from R1

The repeated measures ANOVA showed no main trial effect in plasma triglycerides (*p* = 0.529). Additionally, no significant time-based differences were observed when comparing the I1 vs I2 for PLA (*p* = 0.25) or for CA + C (*p* = 0.993). Both trials demonstrated a time-dependent decrease when evaluating R1 vs R2 (*p* = 0.001, Cohen’s d 0.582; *p* = 0.004, Cohen’s d 0.868), respectively. Means ± SD can be seen in Fig. 4.

### Catecholamines

**Fig. 5:**
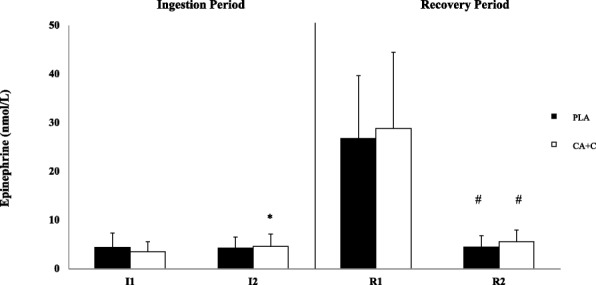
Plasma Epinephrine. Plasma epinephrine is presented as Means ± SD and expressed in nmol/L. * = significantly different from I1. + = Significantly different from PLA. # = Significantly different from R1

**Fig. 6:**
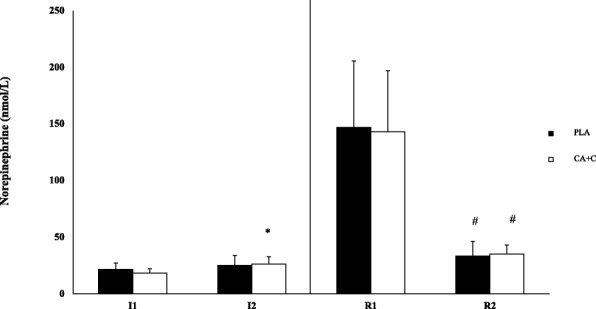
Plasma Norepinephrine. Plasma norepinephrine is presented as Means ± SD and expressed in nmol/L. * = significantly different from I1. + = Significantly different from PLA. # = Significantly different from R1

The repeated measures ANOVA showed no significant trial-based differences between E or NE during the CA + C and PLA ingestion or recovery periods. A significant time-effect was observed during form I1 to 12 only in the CA + C trial for plasma E (*p* = 0.02, Cohen’s d 0.58) and NE (*p* < 0.001, Cohen’s d 1.73). Both CA + C and PLA trials demonstrated time-based decreases between R1 and R2 in E (*p* < 0.001, Cohen’s d 1.74; *p* < 0.001, Cohen’s d 2.00) and NE (*p* < 0.001, Cohen’s d 3.20; *p* < 0.001, Cohen’s d 2.17) respectively. Means ± SD can be seen in Figs. 5 & 6, [15].

## Discussion

The primary purpose of this study was to examine the claim that CA + C supplementation can alter resting metabolic profile, with a secondary aim to evaluate post-exercise metabolic recovery following an exhaustive exercise protocol in habitual caffeine consumers. The major findings of the study indicate that CA + C consumption resulted in a maintenance of glucose levels following the ingestion period, while a significant drop occurred following the consumption of the PLA. No significant trial differences occurred in insulin, lactate or triglycerides throughout the ingestion period. The recovery period for both the CA + C and the PLA trials reveled no trial-based differences.

### Ingestion

The ingestion period of the study occurred over a 45-min time frame in a quiet, relaxed setting following the consumption of CA + C or PLA. Under normal fasted conditions it is not uncommon to observe a slight decrease in blood glucose with concurrent decreases in insulin concentration over a prolong period of rest [17, 18]. Blood glucose concentration following the PLA trial is reflective of this response, with a significant drop occurring at I2. Interestingly, the CA + C ingestion period did not follow this trend. No changes in glucose concentration occurred and was found to be significantly higher than that of the PLA trial at the I2 time point. The glucose response following the CA + C trial is closer to those found by Ratamess et al. [2], who observed a significant increase in concentration following the 45-min ingestion period of p-synephrine + caffeine. However, the Ratamess study observed equivalent changes in blood glucose following the 45-min ingestion periods in the placebo, p-synephrine, and p-synephrine + caffeine trials. This increase in blood glucose could be attributed to the 16 g of carbohydrate used in their supplement compound [2]. The medium by which the supplements were delivered in the current study were capsules absent of carbohydrate and would rationalize the difference in observations between the two studies.

Similar to glucose, insulin has been shown to be maintained or decrease during resting and fasted conditions [19]. The findings in our study are supportive of the expected response, with insulin concentration being maintained in both PLA and CA + C throughout the ingestion period. This is in contrast to Graham et al. [20] who found that caffeine supplementation significantly elevated serum insulin levels when compared to placebo during an oral glucose tolerance test. However, the differences in observations can likely be attributed to the dosage of caffeine Graham et al. [20] used, which was almost five times greater than that of the current study. Though there were no apparent changes in the concentration of insulin following the CA + C trial, several studies have demonstrated that caffeine causes a decrease in insulin sensitivity [21]. This may be a possible mechanism explaining the glucose maintenance observed following the CA + C consumption.

The caffeine components role in sympathetic nervous system (SNS) mediated glucose release [22] may be another likely contributor to the observed glucose response. Following the consumption of CA + C, both plasma E and NE significantly increased, while no changes occurred with the PLA trial. This is in agreement with Graham and Spriet [23], who observed a two-time increase in plasma E following the consumption of 9 mg/kg of caffeine. Additionally, Stuart et al. [24], demonstrated a significantly higher level of plasma E 70-min following the consumption of 6 mg/kg of caffeine when compared to the placebo group. The caffeine concentration given in this trial was a single dose of 100 mg (1.13–1.44 mg/kg), which is far less than the examined dosages of the aforementioned studies. This may in part explain the modest increase in E and NE observed at I2 in the CA + C trial and consequent glucose maintenance as opposed to more robust glucose responses observed in other studies [22]. The CA component of the complex is another mechanism by which the maintenance of blood glucose could have occurred. Specifically, the active ingredient p-synephrine acts on beta-3 receptors in order to increase lipolysis [1], thereby acting to spare blood glucose. However, the findings in our study do not suggest this due to triglycerides remaining unchanged following the consumption of PLA or CA + C. Overall, the present study suggests that the consumption of CA + C in the current dosage appears to promote glucose maintenance at resting conditions. Future research should examine varying concentrations in order to determine a dose effect.

### Recovery

The exhaustive exercise trial selected for this study was a repeated Wingate protocol designed to induce a high metabolic stress and fatigue. Following the completion of the trials, no differences in glucose, insulin, triglycerides, or catecholamines were observed. Blood glucose nearly doubled for both CA + C and PLA compared to baseline values, which indicates a normal metabolic response to an acute high intensity protocol [25]. However, insulin did not statistically elevate immediately post-exercise but demonstrated a non-statistical increase at the end of the recovery period. Previous research has demonstrated insulin spikes immediately following prolonged high-intensity protocols [25]; however, the duration of those protocols was ultimately longer than the one used previous studies and may have led to the different insulin response. Though fat oxidation was not directly measured throughout this study, plasma triglycerides were obtained to determine changes in metabolic function. A primary function of the Citrus Aurantium is improved lipid peroxidation through p-synephrine and beta-3 activation, which may alter the release of triglycerides following exercise based on demand, and ultimately influence metabolic recovery. Post-exercise plasma triglycerides have been shown to account for half of the delayed component of excess post exercise oxygen consumption (EPOC) [26, 27], which is a beneficial response to high-intensity exercise. Interestingly, both trials showed spikes in plasma triglycerides at R1 when compared to I2, though no difference was observed between trials. Following the 45-min recovery, both trials demonstrate similar rates in recovery in triglycerides, suggesting that CA + C had no influence.

Though this study was a novel attempt to evaluate the general metabolic responses to the consumption of the CA + C complex, it was not without its limitations. Currently there is no recommended dosage for the CA + C complex, and the current concentrations selected for this study was based on previous research [2]. The primary purpose of the study was to evaluate the CA + C complex; however, future research should examine the individual components to determine a causal relationship. Furthermore, various dosages of this complex should be evaluated in order to better determine a dose-response effect. The markers used to examine metabolism were glucose, insulin, and triglycerides; future research should examine a more extensive metabolic profile including substrate utilization and free fatty acids. Though a priori analysis based on a power of 0.8, alpha level of 0.05, and effect size of 0.3 determined the necessary n size to be 10 [28], a larger population sample should be used in order to better evaluate effects and trends.

## Conclusion

The commercial use of the CA + C supplement is based around the belief that metabolic function is enhanced following its consumption [2]. The findings of this study suggested that normal recommended dosages of 100 mg CA + 100 mg C were sufficient to promote glucose sparing at rest, with modest increases in SNS activity. However, this was not enough to elicit changes in resting insulin, or triglycerides. These findings suggest practical implications of hypoglycemic prevention during prolong (i.e. 45-min) fasted periods. The CA + C complex examined in this study provided a minimal metabolic response following its ingestion; however, the individual role of CA or C in this response cannot be determined. Further research is needed to examine a dose and component response on these metabolic markers.

## Abbreviations

Body Fat: BF%
C: Caffeine
CA + C: Caffeine and citrus aurantium complex
CA: Citrus Aurantium
E: Epinephrine
HCT: Hematocrit
HHQ: Health history questionnaire
NE: Norepinephrine
PAR-Q: Physical activity readiness questionnaire
PLA: Placebo
SNS: Sympathetic nervous system
